# Current Treatment Approach, Emerging Therapies and New Horizons in Systemic Lupus Erythematosus

**DOI:** 10.3390/life13071496

**Published:** 2023-07-01

**Authors:** Panagiotis Athanassiou, Lambros Athanassiou

**Affiliations:** 1 Department of Rheumatology, St. Paul’s Hospital, GR55134 Thessaloniki, Greece; 2 Department of Rheumatology, Asclepeion Hospital, Voula, GR16673 Athens, Greece; lambros.ath@gmail.com

**Keywords:** systemic lupus erythematosus, treatment, hydroxychloroquine, corticosteroids, biologic agents, belimumab, rituximab, anifrolumab, tocilizumab, baricitinib

## Abstract

Systemic lupus erythematosus (SLE), the prototype of systemic autoimmune diseases is characterized by extreme heterogeneity with a variable clinical course. Renal involvement may be observed and affects the outcome. Hydroxychloroquine should be administered to every lupus patient irrespective of organ involvement. Conventional immunosuppressive therapy includes corticosteroids, methotrexate, cyclophosphamide, mycophenolate mofetil, azathioprine, cyclosporine and tacrolimus. However, despite conventional immunosuppressive treatment, flares occur and broad immunosuppression is accompanied by multiple side effects. Flare occurrence, target organ involvement, side effects of broad immunosuppression and increased knowledge of the pathogenetic mechanisms involved in SLE pathogenesis as well as the availability of biologic agents has led to the application of biologic agents in SLE management. Biologic agents targeting various pathogenetic paths have been applied. B cell targeting agents have been used successfully. Belimumab, a B cell targeting agent, has been approved for the treatment of SLE. Rituximab, an anti-CD20 targeting agent is also used in SLE. Anifrolumab, an interferon I receptor-targeting agent has beneficial effects on SLE. In conclusion, biologic treatment is applied in SLE and should be further evaluated with the aim of a good treatment response and a significant improvement in quality of life.

## 1. Introduction

Systemic lupus erythematosus (SLE), the prototype of autoimmune diseases, is a highly heterogenous disease that affects all organ systems and has an unpredictable course [1]. Its course ranges from mild to severe or fatal disease [2]. Women within the reproductive period are mainly affected [3]. Females originating from Africa or Asia are frequently affected and may exhibit severe disease manifestations [3]. SLE treatment is the focus of scientific research, as biologic agents and small molecules enter management of the disease [4].

SLE follows a variable and unpredictable course [5]. It can be chronic or follow a relapsing and remitting course. Individuals with the disease may present with serious musculoskeletal, cardiovascular and ocular manifestations [6]. Many of these symptoms are disease manifestations or may be caused by the application of corticosteroids for disease management [7]. In 2014, the principle “treat to target” was introduced into the treatment strategy of SLE [8]. Flares, target organ involvement, refractory disease, adverse effects of conventional immunosuppressive agents and a better understanding of molecular pathogenesis of SLE as well as the availability of biologic agents led to the application of biologic agents and small molecules in SLE treatment [9,10]. New biologic agents are in development for the management of SLE with various molecular therapeutic targets. Hydroxychloroquine is the standard mainstay treatment for SLE, and it is recommended by EULAR for patients with symptoms but without major organ lesions [11]. Corticosteroids are administered for the treatment of SLE, but they induce broad immunosuppression, and their use is accompanied by side effects. Hence, it has been proposed that glucocorticoid use should be limited to the shortest possible time and the lowest dose possible. For patients who do not respond to hydroxychloroquine and whose glucocorticoid dosage cannot be reduced, azathioprine and methotrexate are recommended. For patients with severe organ lesions and high disease activity, the use of immunosuppressive agents, pulse cyclophosphamide and mycophenolate mofetil may be applied in combination with hydroxychloroquine. Mycophenolate mofetil may be indicated if there is target organ involvement, such as renal, lung or brain involvement. As standard immunosuppressive treatment is non-specific, biologic agents have been developed and several others are in development with the aim of achieving targeted immunomodulation, disease remission and improved quality of life. Belimumab, an anti-B cell biologic, has been approved for the management of SLE. Rituximab, an anti-CD20 antibody targeting B lymphocytes, is also used in severe SLE. Anifrolumab, an interferon I receptor antagonist, has been applied successfully in the treatment of SLE. In the present review, the therapeutic management and the introduction of biologic agents and small molecules in SLE treatment, along with recent progress and new horizons within the field, will be discussed.

## 2. Methods

**Figure 1:**
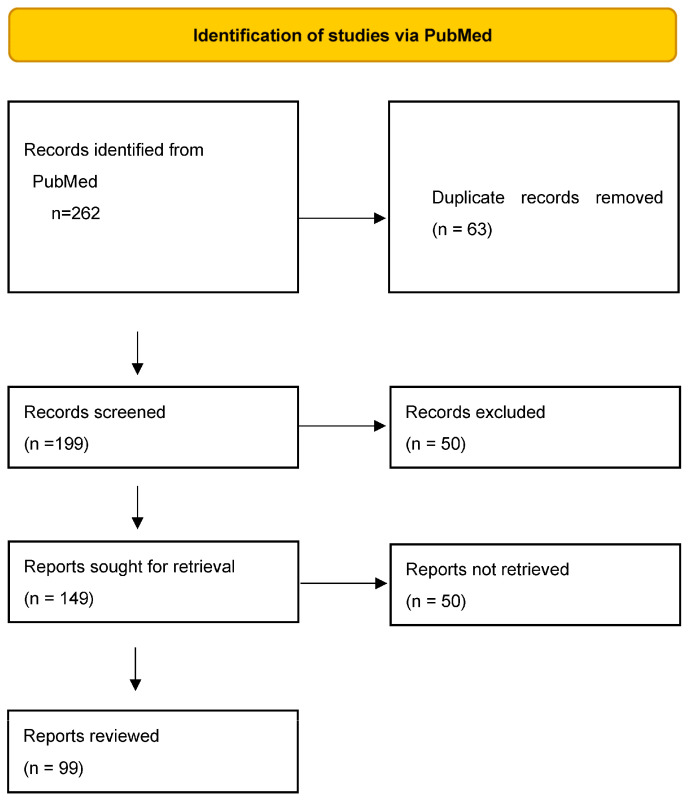
Search method based on the PRISMA flow diagram for identifying studies regarding biological treatment in SLE.

All articles published in PubMed regarding treatment in SLE in the period from 2000 to 2022 were reviewed. Articles reviewed included clinical trials and reviews. The search was limited to articles in the English language. After the elimination of duplicates, eligible articles were read and evaluated (Figure 1). The search methodology was that of the PRISMA 2020 flowchart [12].

## 3. Systemic Lupus Erythematosus Treatment

### 3.1. Hydroxychloroquine

Hydroxychloroquine, an antimalarial, when taken by soldiers during the Second World War for the prevention of malaria, was shown to improve musculoskeletal complaints. Thus, it was utilized in the treatment of rheumatic conditions. Hydroxychloroquine is now considered the standard-of-care treatment of SLE, as it was shown to significantly reduce mortality in all ethnic groups [13,14,15], unless there are contraindications to it [16,17]. It is utilized in the treatment of discoid lupus and SLE [18]. Hydroxychloroquine modulates the immune response by modulating macrophage and other antigen presenting cell function [19,20] and by blocking Toll-like receptors on dendritic cells [21]. Hydroxychloroquine prevents lupus flares, increases survival in all population groups and decreases lupus activity in pregnancy without adverse effects on the fetus [22]. Hydroxychloroquine may also prevent irreversible organ damage, bone destruction and thrombosis [23]. Hydroxychloroquine reduces disease activity in SLE, improves lipid levels and prevents subclinical atherosclerosis [24]. The antimalarial agent also improves glucose metabolism [25]. If attention is paid to dosage, antimalarial toxicity is mild, does not occur frequently and is usually reversible. Ruiz-Irastorza et al. declared that hydroxychloroquine is a standard medication in the treatment of lupus patients and that its administration should last for all disease duration [26]. The discontinuation of hydroxychloroquine is performed when retinopathy is suspected or documented. Its discontinuation may improve the electroretinogram in cases of suspected retinopathy [27].

### 3.2. Glucocorticoids

Glucocorticoids are used in SLE at every dose level, including large, medium and small doses. Large bolus doses may be used as needed in cases of disease flare or target organ involvement and small doses as maintenance treatment [28] to reduce disease activity and disease burden accumulation. They act via a genomic pathway, involving transrepressive and transactivating modes of action on the cell nucleus, and via a non-genomic pathway [29]. However, they induce broad immunosuppression, and their use is accompanied by side effects. Therefore, it has been suggested that their use should be limited in time and dose as much as possible [30,31].

### 3.3. Azathioprine

Azathioprine is administered in SLE as a conventional immunosuppressive agent that aids in steroid sparing. It may be administered as maintenance treatment in renal disease in lupus [32] and in lupus flares. Its administration is safe during pregnancy but unsafe during lactation [33].

### 3.4. Methotrexate

If low dose glucocorticoids do not control the disease, methotrexate may be applied as an immunosuppressive agent, which contributes to steroid sparing [34,35]. Methotrexate has an antifolate mechanism of action [36]. It is indicated in lupus patients who display an inadequate response to hydroxychloroquine and in patients with cutaneous and articular involvement [37] without renal disease. Methotrexate enters cells through a folate transporter [35]. Once within the cell, methotrexate as monoglutamate forms polyglutamates, a more potent drug form which inhibits various enzymes, leading to increased adenosine levels [38], the decreased production of ammonium and H_2_O_2_ and decreased synthesis of purines, methionine and DNA. Adenosine is a molecule with anti-inflammatory effects [39]. Methotrexate is administered in moderate or severe lupus, which does not respond to hydroxychloroquine, with the aim of steroid sparing. It has been shown to reduce disease activity in lupus patients as well as allowing the reduction in glucocorticoid dosage [40] and being beneficial to patients with articular and cutaneous involvement [41]. It is teratogenic and it should be withdrawn before conception [42].

### 3.5. Mycophenolate Mofetil

Mycophenolate mofetil (MMF) inhibits inosine 5-monophosphate dehydrogenase, thereby inhibiting the synthesis of guanine. Thus, B cells, T cells and fibroblasts are decreased. MMF also reduces transforming growth factor β and fibronectin synthesis, thereby exhibiting antifibrotic effects [43]. MMF inhibits the expression of cell adhesion molecules, thereby interfering with the recruitment of lymphocytes and monocytes in the sites of inflammation. It may also induce T cell apoptosis [44]. The first trial in lupus with MMF was performed in 2000. Thereafter, MMF became a standard drug for the treatment of lupus nephritis [45]. It is also used in non-renal lupus. Good quality studies performed in patients with lupus nephritis have proven that MMF is equivalent to IV cyclophosphamide and equivalent or superior to azathioprine during maintenance treatment [46,47,48,49,50]. The beneficial effects of MMF are observed in patients with hematological involvement, refractory cutaneous manifestations and arthritis. MMF is less toxic than cyclophosphamide. It displays gastrointestinal side effects, suppresses the bone marrow and increases the risk of infection. Long-term cancer risk is increased due to its immunosuppressive action. It should be said that there are subgroups of patients with specific susceptibility to the agent, such as an Asian subgroup who are extremely sensitive to MMF if combined with high-dose glucocorticoids [51,52,53]. In African-American patients, who are at high risk for the development of renal involvement, MMF effectively prevents the exacerbation of renal lupus [54].

### 3.6. Cyclophosphamide

Cyclophosphamide is an alkylating drug which acts on DNA and leads to the death of activated lymphocytes while simultaneously having a protective effect on glomeruli [55,56]. Cyclophosphamide displays side effects such as leukopenia, an increased infection risk, bladder toxicity and an increased cancer risk [57]. Cyclophosphamide may be applied as induction therapy for flares or target organ involvement [53,58]. Thereafter, it is transitioned to maintenance treatment with a different drug.

### 3.7. Calcineurin Inhibitors

Calcineurin inhibitors tacrolimus and cyclosporine have been applied as immunosuppressives in organ transplantation. They modulate the immune response mainly by inhibiting T cell activation. Additionally, they reduce albuminuria and preserve renal function [59]. In SLE without renal involvement, cyclosporine contributes to lowering steroid dosage, reduced disease activity and flare prevention [60] by modulating T cell function [61,62]. Tacrolimus can be combined with MMF and steroids as induction therapy in renal lupus with beneficial effects [63,64,65]. It is also used successfully in refractory lupus nephritis [66]. However, serious adverse events can be observed, such as infections and diabetic metabolic derangement. Cyclosporine is also successfully applied in non-responsive to treatment lupus nephritis, with main adverse events observed tremor and hypertension [66]. Voclosporin is a new calcineurin inhibitor, which has been approved for the treatment of lupus nephritis [67,68]. Voclosporin co-administered with MMF and low dose steroids led to more patients achieving a complete renal response as opposed to the combination of MMF with steroids [69].

### 3.8. Intravenous Immunoglobulin

Therapeutic intravenous immunoglobulin (IVIg) is a product which contains human multi-specific immunoglobulin G. IVIg has been used successfully in lupus patients leading to a reduction in disease activity [70]. IVIg was shown to be effective for various manifestations in SLE. It was shown to be effective for renal disease in SLE, as well as target organ manifestations, such as thrombocytopenia, refractory neuropsychiatric lupus [71] and lupus myocarditis [72]. IVIg may act via various mechanisms, including the inhibition of autoreactive B lymphocytes [73]. IVIg is a safe and beneficial mode of treatment for patients with SLE [74] who are resistant to or refuse other types of treatment.

## 4. Biologic Treatment in Systemic Lupus Erythematosus

Flare occurrence, target organ involvement, the inadequate response of some SLE patients to conventional immunosuppressive treatment and the side effects of broad immunosuppressives have led to the application of biologic agents in SLE treatment (Table 1) [9]. The increasing and deeper study of disease pathogenesis has contributed to the introduction of biologic agents to the treatment of lupus [75]. Agents targeting various pathogenetic paths have been applied and others are being studied [9,28]. In particular, B cell targeting agents, interferon targeting agents, tumor necrosis factor a (TNFa) inhibitors and other biologic agents are being investigated.

**Figure 2:**
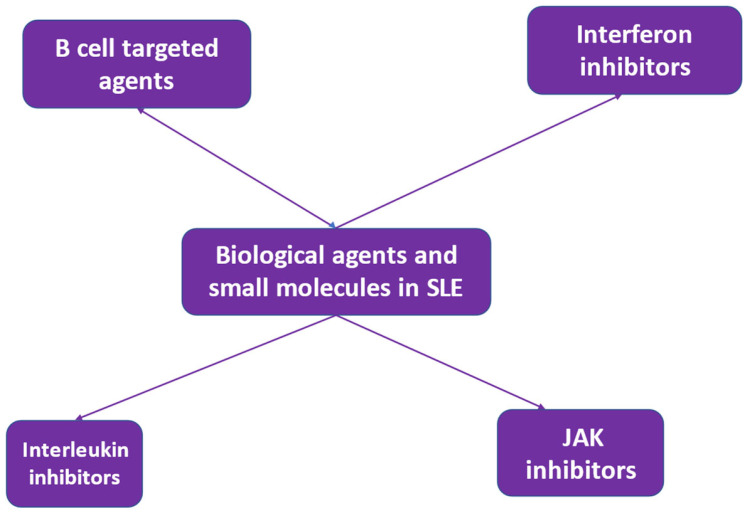
Biologic agents and small molecules in the treatment of systemic lupus erythematosus (SLE).

Biologic drugs that are in use and display beneficial effects in SLE include rituximab [76,77,78] and belimumab [79,80,81,82]. The use of rituximab followed by belimumab is also being investigated [83,84]. Other B cell-targeting biologic agents are also being studied [85]. A variety of biologic drugs targeting various molecular pathways have been introduced in treatment schedules for SLE with refractoriness or intolerance to standard-of-care treatment [86] (Figure 2). The aim of the introduction of biologics into SLE treatment is the achievement of disease remission and the establishment of self-tolerance [87,88]. This aim has not yet been reached. Further research and a deeper understanding of disease heterogeneity and molecular mechanisms involved in SLE pathogenesis may lead to the development of agents targeting specific pathogenetic pathways, which may be efficacious in specific groups of SLE patients [86].

### 4.1. B Cell Targeted Treatment

**Figure 3:**
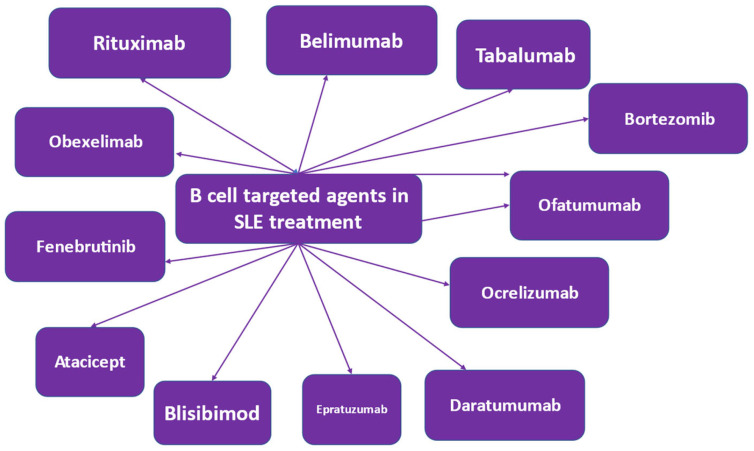
B cell-targeted biologic agents in the treatment of systemic lupus erythematosus (SLE).

SLE pathophysiology is characterized by B cell involvement [89,90]. Therefore, various therapeutic strategies targeting the B cell have been applied [91,92]. B lymphocytes are involved in antibody-dependent and antibody-independent mechanisms in SLE pathogenesis. Autoantibodies are produced by B cells, which are self-reacting, thereby triggering an inflammatory response. In terms of health benefits, B cells produce protective antibodies [93]. In SLE, autoantibodies are produced, which are involved in triggering an inflammatory response via multiple mechanisms, including the induction of cytokine and interferon production by innate immune cells [94]. This immune mechanism is disturbed in SLE patients and is further disrupted by the abnormal functioning of other immune cells [95]. Novel treatment methods that target the B cell have been developed or are in development [91,92] (Figure 3). Agents involved in the growth, activation or proliferation of B cells are treatment targets. Additionally, molecules expressed by B cell subpopulations have been discovered, which if targeted, may lead to their depletion, anergy and apoptosis [79,96,97,98,99].

An aim of the management of SLE is steroid withdrawal, as it is accompanied by broad immunosuppression and is fraught with side effects [30]. To this end, the use of targeted immunosuppression has been attempted and has been found to improve outcomes for some, but not all, patients and with some, but not all, agents applied [28,100]. B cells express various cell surface antigens, depending on the stage of maturation. B cells, mature and immature, express CD20 and CD22, which are B cell surface antigens. These surface antigens are not expressed by plasma cells. It has been suggested that SLE treatment failure in patients administered agents targeting B cell surface antigens may be due to plasma cells, which do not express CD20. This led to the application of alternative B cell targets like the B lymphocyte stimulator (BlyS), known as the B cell-activating factor (BAFF) and the proliferation-inducing ligand (APRIL), which are members of the TNF cytokine group and are applied as targets in the treatment of SLE. Elevated BlyS levels are found in the circulation of lupus patients and are associated with disease activity. These data led to the identification of BlyS as a target for SLE treatment. Intracellular signaling molecules involved in B cell activation include Bruton’s tyrosine kinase. The inhibition of Bruton’s tyrosine kinase is investigated in SLE therapy [101,102]. A proteasome inhibitor, which specifically inhibits B cell differentiation, has also been studied and works via a toxic effect on plasma cells.

#### 4.1.1. Rituximab

Rituximab is a B cell-depleting anti-CD20 monoclonal antibody, applied as a B cell-targeted treatment. Rituximab depletes CD20-positive B cells; however, it spares stem cells and plasma cells, as they do not express the CD20 molecule [103,104]. Rituximab depletes B cells via antibody-dependent and complement-mediated cytotoxicity [105]. It induces B cell apoptosis and reduces proliferation. Rituximab may be used to treat refractory SLE with renal and neuropsychiatric manifestations [106,107,108]. In a comprehensive review, rituximab was shown to induce a significant improvement in systemic manifestations in >90% of lupus cases [109]. Rituximab has not yet been approved by the FDA for SLE treatment, as some trials failed to achieve their primary endpoints [110].

Rituximab has been evaluated in the treatment of SLE in randomized controlled trials and the EXPLORER and Lupus Nephritis Assessment trials. Rituximab was found to be effective in refractory SLE [111,112], safe and effective in non-renal SLE [112] and effective in refractory neuropsychiatric SLE [113]. Rituximab reduces disease activity and immunologic parameters and contributes to lowering steroid dosage. It is beneficial in the treatment of arthritis and thrombocytopenia. In comparison to MMF and cyclophosphamide, it has been shown to be equally effective in renal disease in lupus [114]. Incomplete B cell depletion is observed as CD20 is not expressed by early B cells and plasma cells [115]. Rituximab normalizes B cell subsets in SLE patients [106]. Previously, it was thought that complete B cell depletion might bring about a better outcome for SLE [106]. However, SLE flares were observed after repeated rituximab administration. Flares were attributed to elevated circulating CD257 (BLyS) levels and increased anti-dsDNA levels [116,117]. Hence, it was proposed that rituximab administration acting via B cell depletion might have paradoxically increased CD257 levels, thereby leading to higher SLE activity [115]. Rituximab B cell depletion paradoxically induced peripheral B cell reconstitution and an increase in plasmablasts, which may lead to T helper cell stimulation, autoantibody production and an augmentation in disease activity [115]. Thereafter, rituximab is contemplated for introduction in the management of lupus nephritis only after the failure of conventional immunosuppressants or in relapses of lupus nephritis [118]. Rituximab was shown to be effective in class V lupus nephritis but not in proliferative disease [110,119].

#### 4.1.2. Belimumab

Belimumab is a fully humanized monoclonal antibody against BlyS. It has been administered in clinical trials intravenously and subcutaneously. Belimumab was approved by the FDA for the treatment of seropositive, moderate SLE. Two international clinical trials [79,120] in autoantibody-positive adult patients with active SLE evaluated belimumab. SLE patients were randomized to receive either belimumab or placebo in addition to standard-of-care treatment. Both trials reached their primary endpoints and demonstrated reduced SLE disease activity and flares. The analysis of these trials documented that the rate of lupus disease flares, serologic activity and steroid dosage were significantly reduced, while quality of life was improved by belimumab plus standard-of-care SLE therapy [121]. Belimumab applied subcutaneously also displayed good efficacy, as it decreased disease flares and enabled steroid sparing. Belimumab has also been approved by the FDA for the treatment of autoantibody-positive moderate SLE in children. Belimumab is an anti-CD257 monoclonal antibody and was the first agent to be approved by the FDA for SLE over a period of time of more than 50 years [122,123,124,125,126,127,128,129]. Belimumab should be contemplated in lupus patients without renal involvement, responding inadequately to hydroxychloroquine, glucocorticoids and immunosuppressants [130]. A better response is observed in patients with cutaneous and musculoskeletal involvement. Belimumab has been shown to decrease albuminuria and improve neuropsychiatric manifestations in lupus [131]. Although belimumab is indicated for lupus patients without renal involvement, there are now studies showing promising results in lupus nephritis [120]. In lupus nephritis, belimumab treatment led to a primary efficacy renal response and complete renal response in patients [120]. Belimumab lowered the risk of death related to kidney involvement. The sequential use of rituximab followed by belimumab has also been attempted in several trials [132,133], as well as the concurrent use of belimumab and rituximab, with promising results [134], showing a possible synergistic effect [83].

#### 4.1.3. Tabalumab

Tabalumab, is a monoclonal antibody against soluble and membrane-bound BlyS [135]. Tabalumab was tested in two phase III trials, namely ILLUMINATE-1 and ILLUMINATE-2, in adult patients with moderate to severe SLE without kidney involvement [97,135]. ILLUMINATE-1 did not meet the primary efficacy endpoint in the tabalumab arm—as opposed to ILLUMINATE-2, in which the SRI_5 response was met—in the cohort receiving tabalumab 120 mg twice monthly. Depression was observed in some patients on tabalumab, and some patients attempted suicide.

#### 4.1.4. Atacicept

Atacicept is an antagonist of BlyS- and APRIL-mediated B cell activation. It is a fused protein of the TACI (transmembrane activator calcium moderator and cyclophilin ligand interactor) and IgG, which binds to both Blys and APRIL. As both BlyS and APRIL have been found to be increased in SLE patients, it was suggested that the dual blockade by atacicept might be more effective than BlyS blocking [98]. Atacicept was tested in a phase II b study in SLE patients [136]. However, it did not meet the primary endpoint of the trial in any of its arms, although in a secondary analysis of patients with active disease, atacicept was found to meet primary endpoints and to decrease flare risk. However, deaths due to alveolar hemorrhage in the context of pneumonia were noted in the atacicept arm of the trial.

#### 4.1.5. Blisibimod

Blisibimod is a moiety that inhibits BlyS and displays the characteristics of a peptide and an antibody. It was tested in a phase II trial in SLE and did not reach the primary efficacy endpoint of SRI-5 response [137]. A beneficial treatment effect was observed in SLE patients with high disease activity, where it appeared to be effective in lowering the steroid dosage [138]. The drug appeared to be well tolerated with no serious reported adverse events and no deaths in the treatment arms.

#### 4.1.6. Epratuzumab

Epratuzumab is a monoclonal antibody against CD22. It binds to CD22, thereby inhibiting B cell activation [139,140]. CD22 is a molecule, which is expressed on mature B cells, not on plasma cells or memory B cells, and acts as an inhibitory co-receptor of the B cell receptor and modulates B cell activation and migration. A failure in medication supply led to the premature termination of epratuzumab phase II and III trials. Consequently, two phase III clinical studies, namely EMBODY-1 and EMBODY-2, indicated initial rapid improvement in SLE patients but they did not achieve their primary efficacy endpoint [141].

#### 4.1.7. Daratumumab

Daratumumab is a monoclonal antibody against CD38, a molecule expressed on plasmablasts [142]. CD38 is expressed on plasmablasts, CD19+ mature B cells and plasmacytoid dendritic cells in lupus patients. Daratumumab was administered to two female patients with lupus nephritis and autoimmune hemolytic anemia not responding to immunosuppression [143]. Patients were administered belimumab after the unsuccessful administration of daratumumab.

#### 4.1.8. Ocrelizumab

Ocrelizumab is a fully humanized monoclonal antibody against CD20 with higher antibody-dependent complement and lower complement-dependent cytotoxicity effects as compared to rituximab in SLE patients [144]. Ocrelizumab was successfully used in relapsing, remitting and primary progressive multiple sclerosis [145]. Ocrelizumab was tested in patients with lupus nephritis with beneficial effects, but it displayed a high rate of serious infections [146].

#### 4.1.9. Obinutuzumab

Obinutuzumab, a novel humanized type II glycoengineered anti-CD20 antibody, is a B-cell targeting treatment, which may be administered in SLE patients [142,147]. Studies performed in vitro indicated that obinutuzumab may induce higher B cell cytotoxicity as compared to rituximab in SLE [147,148]. Thereafter, it was suggested that in lupus patients not responding to rituximab, obinutuzumab might be a choice [149].

#### 4.1.10. Ofatumumab

Ofatumumab is a fully humanized anti-CD20 monoclonal antibody. It has been applied as a B cell-depleting agent, which may be used in patients with SLE intolerant to rituximab, i.e., in patients who develop infusion reactions to rituximab [150,151]. It induces antibody- and complement-dependent cytotoxicity in B lymphocytes expressing CD20. Ofatumumab exhibits potency in B cells lysis, which stems from its ability to bind with high affinity to the short extracellular part of the CD20 molecule and its slow release from the target molecule. Ofatumumab was used in an SLE patient with low complement levels combined with fresh frozen plasma [152]. Lupus patients intolerant to rituximab infusion may be administered ofatumumab [150]. It is well accepted and may represent an alternative B cell-targeted treatment in SLE.

#### 4.1.11. Obexelimab

Obexelimab is a humanized anti-CD19 monoclonal antibody targeting FcgRIIb, which is a reversible B-cell inhibitor [142]. CD-19 is a cell surface molecule found on B cells, plasmablasts and plasma cells [153]. It was hypothesized that targeting CD19 could lead to significant B cell and plasma cell depletion in lupus patients. Obexelimab was tested in a phase II randomized trial in moderately active lupus patients. However, it did not reach its primary endpoint and further studies were not conducted.

#### 4.1.12. Bruton’s Tyrosine Kinase-Targeted Treatment

Currently, B-cell signaling is a target for B-cell treatment in SLE. Tyrosine kinases, Bruton’s tyrosine kinase in particular, acts as an intracellular molecule essential for the development, survival and activation of B cells. Bruton’s tyrosine kinase is involved in antigen presentation, B-cell differentiation and the production of autoantibodies in SLE [154]. In experimental animal models, Bruton’s tyrosine kinase inhibition was shown to have beneficial effects in SLE [155].

In a phase II trial, fenebrutinib, a highly selective oral inhibitor of Bruton’s tyrosine kinase, was tested in moderate to severe SLE in addition to standard-of-care treatment [102]. The trial did not reach its primary endpoint; however, beneficial effects of fenebrutinib in SLE were observed. Ibrutinib, another irreversible selective inhibitor of tyrosine kinase, was tested in lupus nephritis mouse models [156].

#### 4.1.13. Proteasome Inhibitors

CD-20 negative cells may be source of treatment failures with CD-20-targeting agents. CD-20 negative cells may be targeted by inhibiting the proteasome. Proteasome inhibition leads to the accumulation of defective immunoglobulin chains and induces stress in the endoplasmic reticulum, leading to plasma cell apoptosis [157]. Bortezomib is a proteasome inhibitor, which has been tested in animal models of lupus [158]. Bortezomib was also tested in SLE patients. Bortezomib was tested in severe refractory lupus nephritis [159] and exhibited good results in SLE [157,160]. However, severe side effects observed led to the idea that it might be used only as salvage treatment for refractory lupus patients. Thus, bortezomib may be used in SLE patients with very active disease, who have already been treated with rituximab.

#### 4.1.14. Rigerimod

Rigerimod is a peptide which blocks antigen presentation to T cells by reducing the stability of MHC molecules, thereby inhibiting B cell function. Rigerimod has been utilized in lupus patients with encouraging results [161].

### 4.2. Interferon Inhibitors

#### 4.2.1. Sifalimumab

Interferons (IFNs) are immunostimulatory cytokines divided in three categories: types I, II and III [162]. IFNα is a type I IFN that is abundant and has been studied in depth. The role of interferons in the pathogenesis of SLE has been extensively studied and has been proven [163]. Sifalimumab is a fully human monoclonal antibody against IFN-α subtypes and displayed beneficial effects in a phase IIb clinical trial in SLE [164].

#### 4.2.2. Anifrolumab

Type I IFN may be implicated in the pathogenesis of SLE. A gain-of-function genetic mutation in the type I IFN pathway may be associated with a higher risk of SLE [165]. In the period before the clinical presentation of SLE, high type I IFN and SLE autoantibodies have been observed. Patients with established SLE and evidence of high type I IFN may have more active disease and lupus nephritis or other severe manifestations. As reiterated previously within this review, there is a need for new therapeutic modalities in SLE, as existing therapeutic agents have adverse effects. Type I IFNs are mediated by the type I IFN-α/β/ω receptor, known as IFNAR. Anifrolumab, a fully human immunoglobulin G1k antibody, which binds IFNAR, a type I interferon receptor antagonist, showed good results in patients suffering from scleroderma. As similarities have been observed in the type I IFN response between scleroderma and SLE, anifrolumab was tested in patients with SLE. The MUSE (MEDI-546 in Uncontrolled Systemic Lupus) trial was implemented to assess the safety and efficacy of anifrolumab. This trial was followed by the phase III trials TULIP-1 (Treatment of Uncontrolled Lupus via the Interferon Pathway-10) and TULIP-2 (Treatment of Uncontrolled Lupus via the Interferon Pathway-2) [166]. These trials led to the approval of anifrolumab by the FDA for moderate to severe SLE, excluding patients with lupus nephritis or central nervous system involvement [167]. It was also approved by EMA in 2022 as an additional treatment for adult patients with moderate to severe SLE, despite conventional treatment. The MUSE trial showed positive effects at its primary endpoint. The TULIP-2 trial also reached its primary endpoint. The most significant adverse effect of anifrolumab was herpes zoster.

### 4.3. Interleukin Inhibitors

#### 4.3.1. Tocilizumab

Tocilizumab is a humanized monoclonal antibody against interleukin-6 receptor [168] and has been mainly used in the treatment of rheumatoid arthritis [169] and is also utilized in the treatment of giant cell arteritis [170]. Tocilizumab has been also used in patients with severe SARS-CoV-2 virus infection [171]. Tocilizumab was used to treat refractory hemolytic anemia in an SLE patient [172]. It has also been used in an adolescent patient with SLE and a pleural effusion [173]. It was also used successfully in SLE patients with persistent high grade fever and who were resistant to treatment with antibiotics and corticosteroids [174]. Tocilizumab administration has been shown to have beneficial effects in lupus patients with arthritis [175]. However, neutropenia and risk of infection are factors limiting its use in SLE.

#### 4.3.2. Secukinumab

Secukinumab is a monoclonal antibody which binds to interleukin 17A. It is used in ankylosing spondylitis, psoriasis and psoriatic arthritis [176,177,178]. T-helper 17 cells are thought to be involved in the pathogenesis of SLE [179]. Secukinumab was administered to a female patient with psoriasis and refractory lupus nephritis with beneficial effects [180]. Secukinumab is being evaluated for the treatment of active lupus nephritis [181].

### 4.4. Low Dose Interleukin-2

The loss of immune tolerance characterizes SLE. This loss of tolerance may be due to the impaired function of T regulatory cells (Tregs) [182,183] as well as an imbalance between T follicular helper cells and T follicular regulatory cells. Low dose interleukin-2 in patients with SLE was shown to restore the balance between T follicular regulatory cells and T follicular helper cells in favor of T follicular regulatory cells and display clinical efficacy in SLE [184,185].

### 4.5. JAK Inhibitors

#### Baricitinib

Baricitinib, a selective oral inhibitor of Janus kinase, has been approved for the treatment of rheumatoid arthritis [186]. Baricitinib has been evaluated in active SLE patients not responding to standard-of-care treatment. The resolution of arthritis and rash was observed [187]. However, a high rate of infection was found. A program for the application of baricitinib in the management of SLE was ended as no efficacy was observed.

## 5. Therapeutic Strategies for the Management of SLE

In 2014, the treat-to-target principle was introduced in the strategy for the therapeutic management of SLE [8]. In 2019, the EULAR recommendations based on evidence and expert opinion for the management of SLE were updated [188]. It was concluded that hydroxychloroquine should be administered to all lupus patients. During lupus flares, bolus doses of glucocorticoids should be administered. During maintenance treatment, glucocorticoids should be minimized and, if possible, withdrawn entirely. The initiation of immunomodulatory agents can aid in the reduction or withdrawal of glucocorticoids. However, despite conventional immunosuppressive treatment, flares of the disease occur and some patients may not respond to it. Treatment of flares in unresponsive patients targets organ involvement, and the observation of side effects, the deeper knowledge of pathogenetic paths in SLE and the availability of biologic agents has enabled the introduction of biologic agents and small molecules in the treatment of SLE. B cell-targeting agents have been introduced with success. Rituximab may be used in renal and non-renal SLE. Belimumab has been approved for the treatment of SLE. The sequential use of rituximab followed by belimumab has also been tested in refractory cases. In 2023, the Study Group of Autoimmune Diseases of the Portuguese Society of Internal Medicine issued recommendations for the off-label use of biologic agents and small molecules in SLE [9]. They suggested that in SLE patients with very active disease, i.e., SLEDAI > 20 or BILAG 3A, severe hemolytic anemia, severe thrombocytopenia, severe kidney disease (stage IV) or severe CNS disease, the use of rituximab is recommended as first-line therapy. In patients with very active disease, such as severe kidney disease or severe CNS disease, the sequential use of rituximab followed by belimumab may be applied. In patients with persistently active disease with flares for at least one year or very active disease, rituximab is recommended in rituximab-naïve cases as second-line therapy, and baricitinib or tocilizumab may be used if arthritis predominates as second-line treatment. In lupus patients with severe kidney disease, rituximab is recommended in rituximab-naïve patients as second-line therapy, and the sequential use of rituximab and belimumab may be used in refractory cases as second-line treatment. In patients with very active disease, the sequential use of rituximab followed by belimumab may be applied in rituximab-naïve patients as second-line treatment. In these patients with very active disease, bortezomib may be considered in multi-refractory cases as second-line treatment. In patients with hemolytic anemia or thrombocytopenia, rituximab is recommended and bortezomib may be considered in multi-refractoriness. For moderate or severe CNS disease, rituximab is recommended in rituximab-naïve cases as second-line treatment and the sequential use of rituximab followed by belimumab may be considered in refractory cases as second-line treatment.

## 6. Conclusions

In conclusion, hydroxychloroquine and glucocorticoids are the standard-of-care treatments in the therapeutic management of SLE. When flares occur, conventional immunosuppressive agents are added. Refractory disease, target organ involvement, flare recurrence, the adverse events of conventional immunosuppressive agents and the further understanding of molecular SLE pathogenesis have led to the introduction of biologic agents and small molecules in SLE treatment with the aim of improving disease activity, outcome and quality of life. Target organ involvement may guide the treatment of SLE. The introduction of rituximab and belimumab, biologic agents targeting the B cell, has opened a new era in SLE management and has extended the therapeutic spectrum in all subgroups of lupus patients. New biologic agents and small molecules targeting various pathogenetic paths are in development. Although significant progress has been achieved in SLE treatment, more work is needed to further incorporate therapeutic developments in SLE treatment and to ensure improved quality of life in SLE patients.

## Figures and Tables

**Table 1:** Biologic drugs currently in use in systemic lupus erythematosus.

Biologic Drugs	Mode of Action	Target
Rituximab	B cell targeted	CD20
Belimumab	B cell targeted	BAFF, APRIL
Anifrolumab	Interferon I receptor antagonist	Interferon I receptor
Secukinumab	Antibody to Interleukin 17A	Interleukin 17A
Baricitinib	JAK inhibitor	Janus kinase

## Data Availability

Not applicable.
